# Case Report: Favorable long-term outcomes of autologous PRF-augmented delayed replantation for traumatic avulsion of three mandibular anterior teeth with 10-year follow-up

**DOI:** 10.3389/fdmed.2026.1981309

**Published:** 2026-10-13

**Authors:** Yang Yang, Shuang Qu, Jing Chang, Min Zhang, Yanli Liu

**Affiliations:** 1 National Clinical Research Center for Oral Diseases, State Key Laboratory of Oral & Maxillofacial Reconstruction and Regeneration, Shaanxi International Joint Research Center for Oral Diseases, Department of General Dentistry and Emergency, School of Stomatology, The Fourth Military Medical University, Xi'an, Shaanxi, China; 2 Department of Stomatology, 941th Hospital of People’s Liberation Army Joint Logistic Support Force, Xining, China

**Keywords:** avulsion, delayed replantation, long-term follow-up, periodontal ligament healing, platelet-rich fibrin (PRF)

## Abstract

The first-line treatment for permanent tooth avulsion is immediate replantation. Delayed replantation is associated with a high risk of survival-related complications, such as root resorption. Autologous platelet-rich fibrin (PRF) may promote the periodontal healing of delayed-replanted avulsed teeth and may potentially reduce the occurrence and progression of early root resorption. This case report explores the dental treatment process of autologous PRF-assisted delayed replantation in an adult patient with the avulsion of three mandibular anterior teeth. After an accident, the patient wiped the roots of the avulsed teeth and stored them in dry tissue. Two hours later, the patient visited the emergency department for treatment. On the same day, PRF granules were prepared, and the teeth were replanted with the granules and fixed with semi-elastic splints. The treatment was based on the 2012 guidelines of the International Association of Dental Traumatology. Two weeks later, the patient returned, and root canal treatment was performed, the fixation device was removed at the same visit. Two weeks after intracanal medication with calcium hydroxide, root canal filling was completed. Follow-up visits at 3, 6, and 12 months and up to 10 years after the trauma revealed no clinical symptoms in the affected teeth, and radiography showed no sign of root resorption. The patient expressed satisfaction with both the esthetic and functional outcomes. This case report describes a favorable outcome following PRF-assisted replantation of delayed-replanted avulsed teeth. The findings suggest a potential adjunctive role of PRF in promoting periodontal healing, which warrants further investigation.

## 1 Introduction

Tooth avulsion is defined as the complete displacement of a tooth from its original alveolar socket, and it is among the most severe types of dental trauma. Globally, avulsion of permanent teeth accounts for 0.5%-3% of all cases of dental trauma, whereas some studies have indicated that the global incidence rate can be as high as 16% (1, 2). Generally, maxillary central incisors are the most susceptible to avulsion (3, 4). However, avulsion of mandibular anterior teeth, although relatively uncommon, can also occur in cases of complex trauma, and its long-term outcomes after replantation remain difficult to predict (5).

Tooth avulsion causes severe disruption of the entire tooth structure, with the tearing and disruption of the periodontal ligament (PDL) fibers; the viable PDL cells remaining on the root surface are critical for the long-term retention of the replanted tooth, and their viability is determined by the appropriate storage medium, extraoral storage time, and handling procedures (6). Teeth with an extraoral dry time of more than 60 min have significantly lower survival rates after replantation. Therefore, immediate replantation is the best treatment for tooth avulsion. However, in most cases, delayed replantation is extremely common in clinical practice because of the limited self-treatment ability at the incident site, prolonged tooth exposure to dryness outside the mouth, or the inability to obtain timely dental treatment. Delayed replantation can lead to damage to the dental pulp and the periodontal tissues attached to the root, thereby causing various complications, such as pulp necrosis, root resorption, and even tooth loss (3, 7). Research has indicated that following tooth replantation, the incidence rate of inflammatory root resorption can reach 23%, and the rate of replacement root resorption can reach approximately 51% (3). Ultimately, 21%-89% of replanted teeth are lost, leading to a low and unpredictable long-term survival rate (8). Scientists have made many efforts for improving the clinical outcomes of delayed replantation. Panzarini et al. reviewed treatment strategies involving the application of various agents (e.g., antibiotics, enamel matrix proteins, etching solutions, fluorides, corticosteroids, or bisphosphonates) to the root surface or PDL before replantation; they concluded that, unfortunately, none of the treatments in the reviewed studies could prevent or cure ankylosis or replacement resorption in teeth with severely damaged PDL cells (9).

Platelet-rich fibrin (PRF), a second-generation autologous platelet concentrate, has attracted considerable research attention owing to its ease of preparation and favorable biocompatibility; thus, it has become a highly promising biological adjunct for the treatment of dental trauma. PRF has been widely applied in multiple medical specialties, such as cardiac surgery, oral and maxillofacial surgery, orthopedics, sports medicine, and facial plastic surgery. PRF is prepared by single centrifugation of autologous blood, retains an intact fibrin matrix, and serves as a sustained-release reservoir for growth factors, including platelet-derived growth factor (PDGF) and transforming growth factor-beta (TGF-*β*), which may stimulate angiogenesis, osteogenesis, and periodontal tissue regeneration. PRF has recently attracted attention for its potential to support PDL regeneration and improve post-replantation tissue repair (10). Hiremath et al. reported that PRF may increase the activity of PDL cells *in vitro* (11, 12). Our previous animal experiments suggested that PRF may promote the periodontal healing of delayed-replanted avulsed teeth in dogs, and may help limit the development of early root resorption (13). Case reports have also suggested that for teeth with avulsion injury, PRF may reduce root resorption caused by delayed replantation and may promote PDL healing, thereby improving the outcomes of replantation (14). Nevertheless, the available evidence presents certain limitations. Follow-up durations are generally short (1−2 years), and data on its long-term efficacy are lacking. Moreover, long-term (≥10 years) clinical evidence on the use of PRF as an adjunct to replantation of avulsed teeth is scarce, especially regarding the replantation of multiple avulsed mandibular anterior teeth (15, 16).

This case report describes a patient who underwent PRF-assisted delayed replantation of three mandibular anterior teeth after avulsion. Over the 10-year follow-up period, no complications, such as root resorption, ankylosis, or infection, were observed. This case may help address the gap concerning the long-term outcomes of PRF-assisted multiple-tooth replantation and may provide a reference for clinical practice.

## 2 Case description

### 2.1 Timeline

This case report follows Case Report (CARE) guidelines (17).The overall treatment sequence and long-term follow-up outcomes are summarized chronologically in Figure 1.

**Figure 1:**
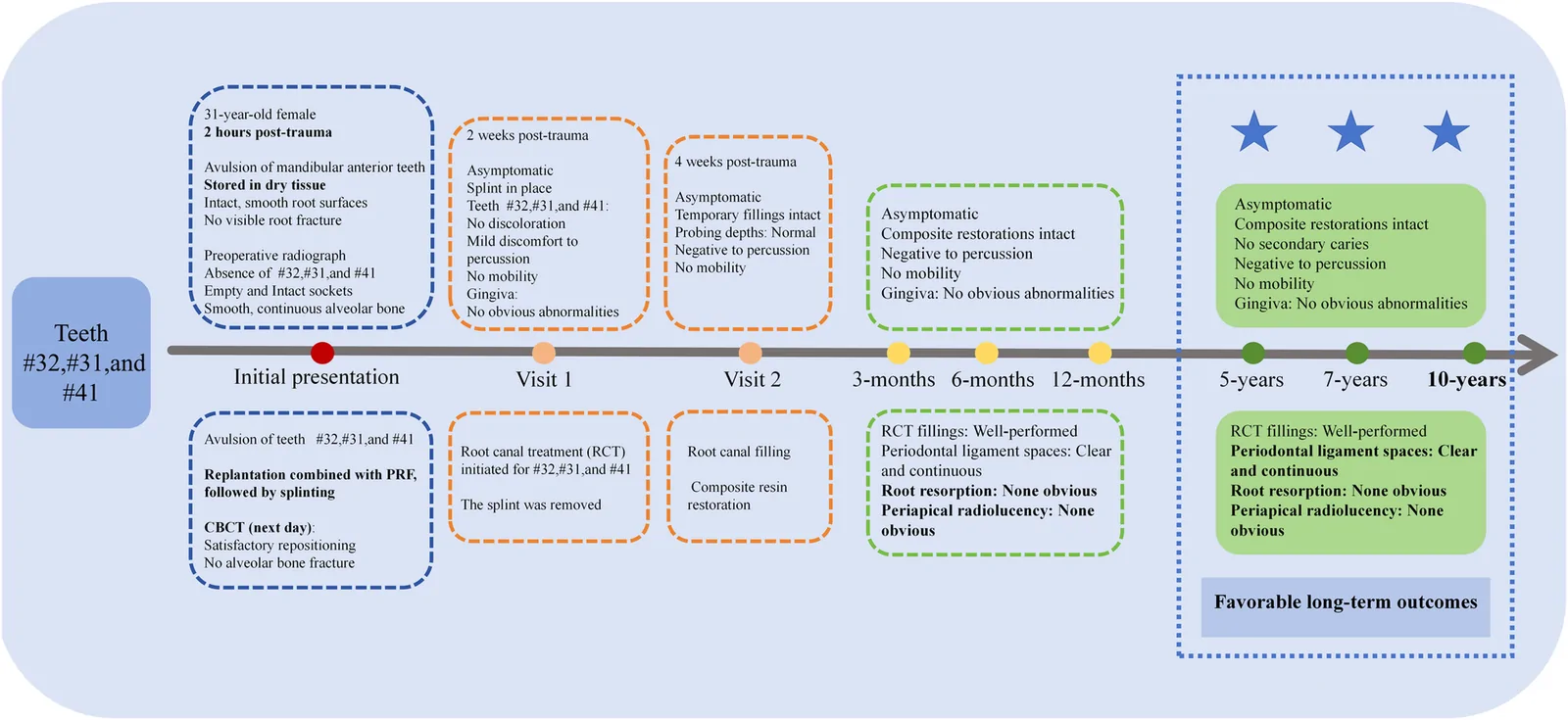
Timeline of the diagnostic findings, traumatic tooth sequential treatment procedures, and follow-up outcomes of teeth #32, #31, and #41.

### 2.2 Clinical and radiological evaluation

A 31-year-old female patient visited the emergency department because of the loss of her front teeth. Two hours ago, the patient had an accident involving impact on a hard object, resulting in the avulsion of the mandibular anterior teeth. She found the avulsed teeth at the accident site, wiped the surface of the tooth roots with a tissue, and then wrapped them in dry tissue for preservation. The patient denied any history of transient loss of consciousness, dizziness, nausea, or vomiting. On presentation, the patient was conscious, had stable vital signs, was in good condition, and was able to communicate independently. The patient was generally in good health, with no reported history of systemic diseases, no history of infectious diseases, and no known drug allergies.

Clinical examination revealed no obvious facial swelling or tenderness. Mouth opening and range of motion were normal, with no clicking, crepitus, or tenderness in the bilateral temporomandibular joints. Overall oral hygiene was fair, with no significant calculus or bleeding on probing and with soft debris and normal gingiva. The right central (#41), left central (#31) and lateral (#32) mandible incisors were missing, with empty sockets containing blood clots; no obvious laceration of the gingiva was observed (Figure 2A). The avulsed teeth had intact roots, no visible root fracture lines, and smooth root surfaces (Figure 2B). An emergency radiograph (FOCUS, Instrumentarium Dental, Finland) revealed missing teeth #32, #31, and #41, empty sockets, smooth and continuous alveolar bone, and intact sockets (Figure 3A). Radiographic assessment at each follow-up was performed using periapical radiographs with the paralleling technique, and the same projection angle was maintained as far as possible to ensure standardization. The assessment included the PDL space, root contour, and periapical bone. Periodontal healing was defined as a normal, continuous PDL space. Inflammatory resorption was defined as an irregular root surface or an external resorption radiolucency. Replacement resorption/ankylosis was defined as direct contact between the root and alveolar bone with loss of the PDL space. Periapical pathology was defined as a periapical radiolucency, loss of the lamina dura, or widening of the periapical ligament space.

**Figure 2:**
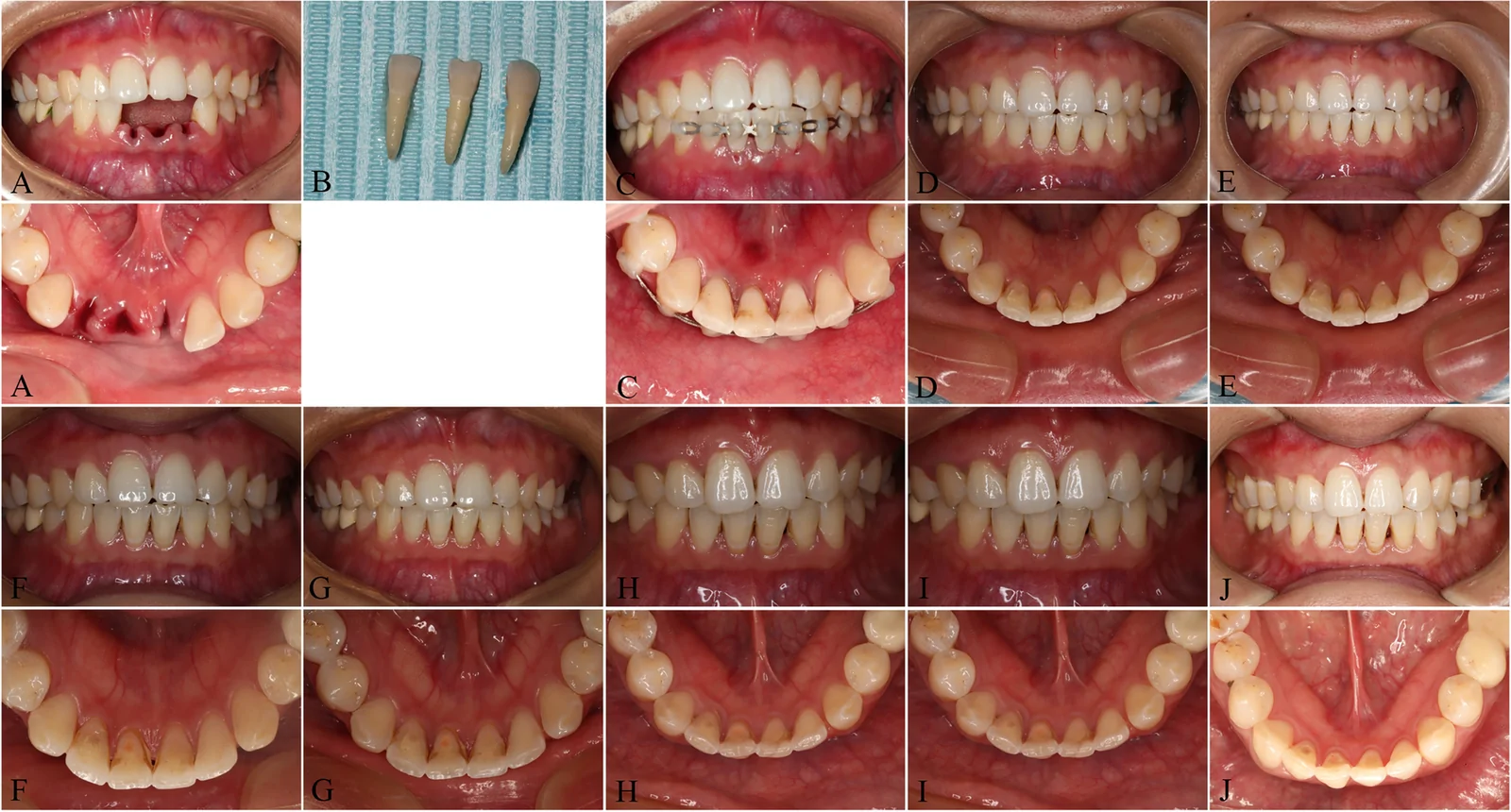
Intraoral images (upper row: frontal view; lower row: occlusal view): **(A)** avulsion of teeth #32, #31, and #41; **(B)** dry-stored avulsed incisors; **(C)** the avulsed incisors were replanted with PRF and splinted; **(D)** root canal filling and coronal composite resin restoration; **(E)** 3-month follow-up; **(F)** 6-month follow-up; **(G)** 12-month follow-up; **(H)** 5-year follow-up; **(I)** 7-year follow-up; **(J)** 10-year follow-up.

**Figure 3:**
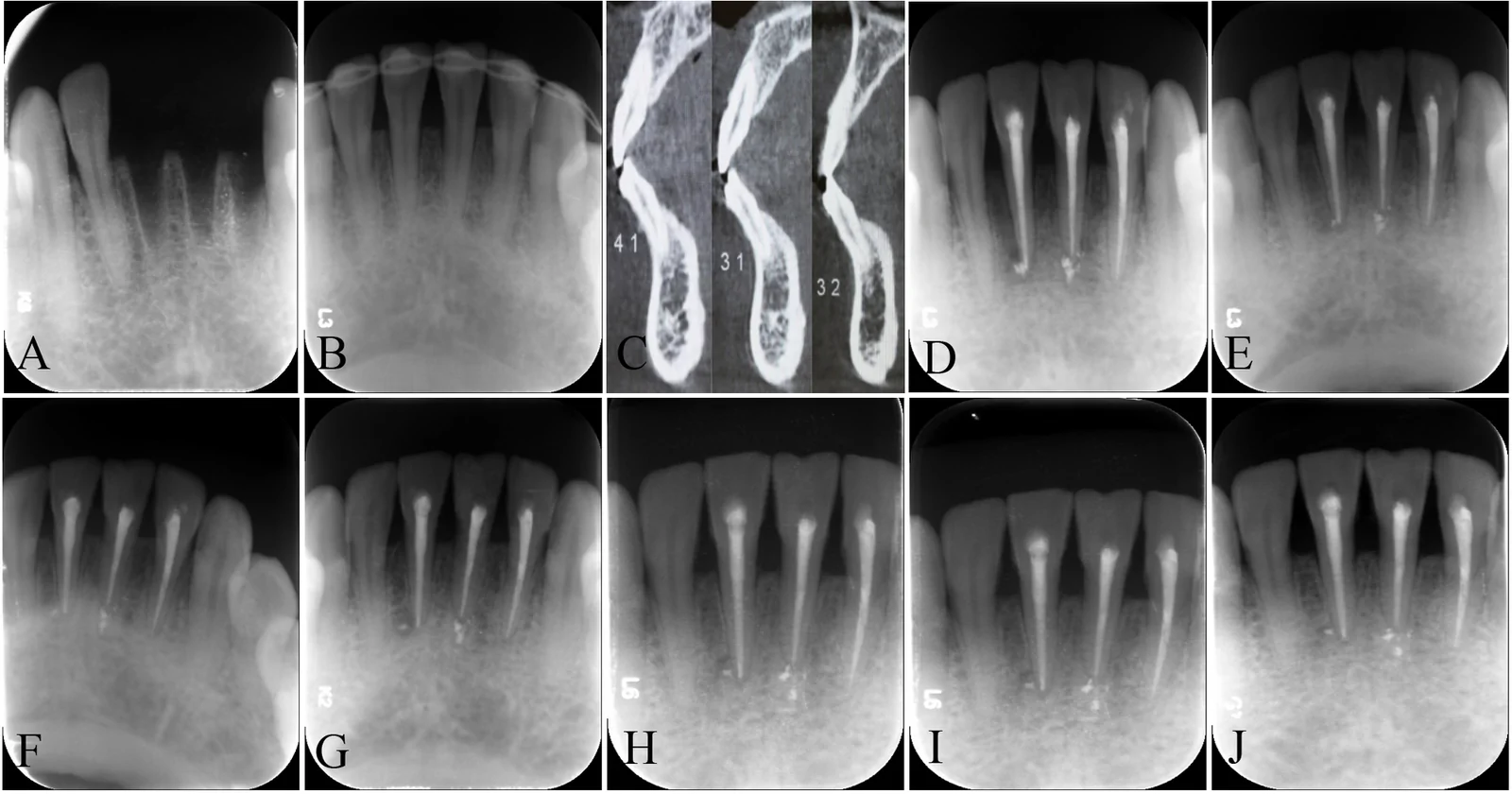
CBCT and radiographic images: **(A)** immediately after the trauma; **(B)** post-splinting; **(C)** CBCT image taken one day post-replantation and splinting; **(D)** post-root canal filling; **(E)** 3-month follow-up; **(F)** 6-month follow-up; **(G)** 12-month follow-up; **(H)** 5-year follow-up; **(I)** 7-year follow-up; **(J)** 10-year follow-up.

The initial diagnosis in the emergency department was avulsion of teeth #32, #31, and #41. Owing to the prolonged extraoral duration of 2 h and the dry storage of the avulsed teeth, the PDL tissue was nearly entirely compromised. Therefore, the expected healing outcome in this case was primarily replacement resorption, rather than PDL regeneration. In this setting, the treatment objective of replantation was not to achieve true periodontal healing, but rather to temporarily retain the teeth, preserve alveolar bone contour, and maintain esthetics and function. Based on preliminary evidence that platelet-derived growth factors in PRF, such as PDGF, TGF-*β*, and vascular endothelial growth factor (VEGF), may promote periodontal tissue remodeling by enhancing fibroblast proliferation, collagen synthesis, and angiogenesis, and may potentially reduce inflammatory root resorption (13, 14, 18), adjunctive PRF was recommended at the time of replantation. However, its effect on ankylosis or true PDL regeneration remains uncertain and warrants further investigation. The patient was informed of the treatment plan, including the estimated time, costs, and prognosis, and subsequently provided written informed consent.

### 2.3 Treatment process

For preparing PRF, 10 mL of venous blood was collected from the patient's antecubital vein, immediately transferred into a 10-mL anticoagulant-free glass tube (Evacuated Blood Collection Tube, Becton Dickinson and Company, Shanghai, China), and centrifuged at 3,000 rpm for 10 min (TD3, CENCE, Changsha, China) (Figures 4A,B). After centrifugation, the blood was left to stand for 5 min and then separated into three layers: the middle layer contained a fibrin clot with PRF, and red blood cells were readily separated in the bottom layer (Figure 4C). The PRF gel was gently grasped with sterile forceps and placed on sterile gauze in a sterile culture dish. Another piece of sterile gauze was placed on top, and the serum was gently squeezed out to obtain a compact autologous fibrin membrane (Figure 4D). The red portion of the membrane was trimmed to approximately 5 mm and cut into granules of approximately 0.5 mm × 0.5 mm using sterile ophthalmic scissors; the red and white portions were mixed evenly for later use (Figure 4E). For teeth #32, #31, and #41, the sockets were examined and irrigated with sterile saline. Blood clots within the sockets were removed without curettage, and the root surfaces were rinsed with sterile saline. No additional local medication was applied to the root surface or socket. PRF granules were placed throughout the sockets using a long-handled instrument to assist replantation, and the teeth were replanted. A preformed semi-elastic titanium alloy lip bow (Titanium Trauma Splint, Zhongbang Titanium Biological Materials Co., Ltd., Xi'an, China) was utilized for fixation (Figure 2C). The tooth surfaces were etched with 37% phosphoric acid, treated with a universal adhesive (3M, USA), and the splint was fixed with flowable composite resin (Shofu, Japan) after its position was confirmed. An immediate postoperative radiograph revealed satisfactory repositioning of teeth #32, #31, and #41 (Figure 3B). Occlusion was adjusted so that the replanted teeth had no occlusal contact with the opposing teeth, and the surfaces were polished. On the second day, a cone beam computed tomography (CBCT) scan (Hires3D, Beijing, China) was performed. CBCT revealed that teeth #32, #31, and #41 were well repositioned, and that no alveolar bone fracture was observed (Figure 3C). Postoperatively, 0.12% chlorhexidine mouthwash was prescribed twice daily for 1 week for oral hygiene maintenance, and the patient was advised to undergo tetanus status assessment. No systemic antibiotics, analgesics, or other medications were prescribed. The patient received postoperative instructions, and a follow-up appointment was scheduled within 7−10 days in accordance with the 2012 guidelines of the International Association of Dental Traumatology(IADT) (19).

**Figure 4:**
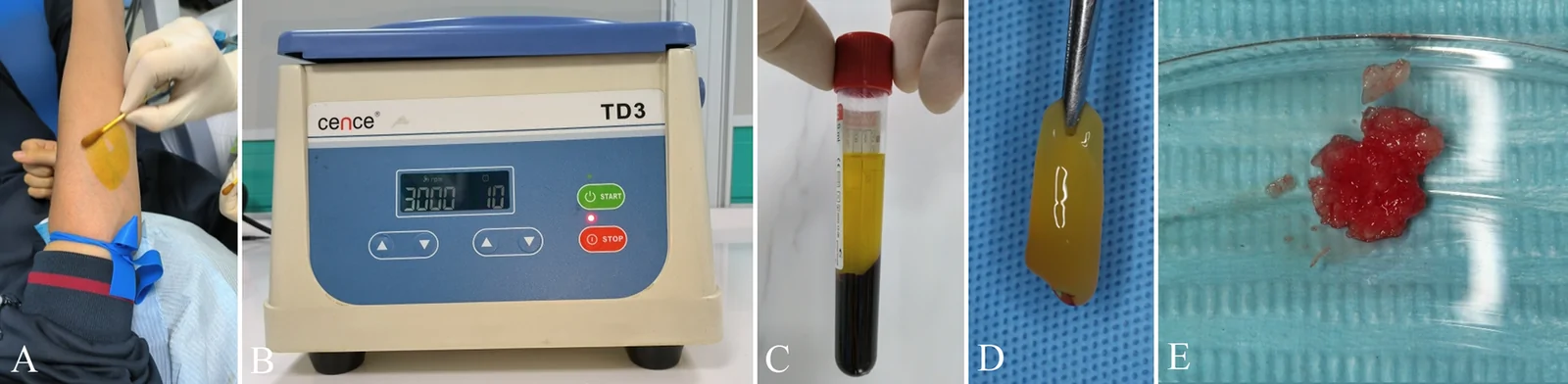
Preparation process of the platelet-rich fibrin: **(A)** Venous blood (10 mL) was collected from the antecubital vein; **(B)** The tube was immediately centrifuged at 3,000 rpm for 10 min; **(C)** The fibrin clot contained platelet-rich fibrin and was located in the middle of the tube; **(D)** The clot was easily separated from the red corpuscles at the bottom; **(E)** The platelet-rich fibrin membrane was cut into granules of approximately 0.5 mm × 0.5 mm.

However, the patient did not return for the scheduled visit and came back approximately 2 weeks later, reporting no discomfort in the avulsed teeth between appointments. Clinical examination revealed the appropriate placement of the splint. Teeth #32, #31, and #41 showed no discoloration, percussion elicited mild discomfort without mobility. No obvious abnormalities of the gingiva were noted. Root canal treatment was initiated for teeth #32,#31,and #41. Under rubber dam isolation, straight-line access was prepared. The pulp was extirpated, and a single canal was identified in each tooth under the microscope (Zumax OMS2350, Zumax Medical, Suzhou, China). The canals were explored and negotiated with K-files to establish apical patency and a glide path. The working length (WL) was determined with an apex locator (VDW Gold, MTwo VDW, Germany), set 0.5 mm short of “0” and radiographically confirmed. The working length was 17 mm for tooth #32 and 18 mm for teeth #31 and #41. EDTA gel (Glyde, USA) was used to lubricate the canals. Shaping was performed with ProTaper Gold (TG) rotary nickel-titanium instruments (Dentsply Sirona, Charlotte, NC, USA) using a crown-down technique; the final apical preparation size and taper were 35#/.06. During instrumentation, the canals were irrigated with 2.5% NaOCl after each file change. After instrumentation, the canals were irrigated with 3 mL of 2.5% NaOCl left *in situ* for 4 min and ultrasonically activated (Satelec, Acteon Group, Mérignac, France). The canals were dried with paper points, filled with calcium hydroxide paste (Calcicur/Voco, Germany), and temporarily sealed with Cavit (3M ESPE, USA). Given the absence of alveolar bone fracture, the splint was removed in the same visit.

The patient returned for a follow-up visit 2 weeks after medication installation, and she reported no discomfort in the involved teeth between appointments. Clinical examination revealed the intactness of the temporary fillings in teeth #32, #31, and #41, with normal probing depths. The response to percussion and mobility testing was negative. After removal of the temporary filling, the calcium hydroxide paste was flushed from the canals with sterile saline. The canals were then irrigated with 2.5% NaOCl activated by ultrasonic K-file agitation, followed by drying under rubber dam isolation. A cone-fit radiograph was taken to confirm the fit of the master cone. Obturation was completed with large-taper gutta-percha (Dentsply, USA) and iRoot SP sealer (Septodont, France) using warm vertical compaction for the apical 5 mm, followed by thermoplastic gutta-percha backfilling of the coronal portion of the canals. The access cavities of teeth #32, #31, and #41 were restored with composite resin (3M ESPE (Solventum), St. Paul, MN, USA) (Figure 2D; Figure 3D).

### 2.4 Follow-u*p* and outcomes

At the 3-, 6-, and 12-month post-trauma follow-up visits, the patient reported good subjective well-being and no discomfort. Clinical examination revealed the intactness of the composite resin restorations on teeth #32, #31, and #41, with no tenderness to percussion and no mobility, as well as no obvious abnormalities of the gingiva (Figures 2E–G). Radiographic examination revealed satisfactory root canal fillings in teeth #32, #31, and #41, with clear and continuous PDL spaces and no obvious radiographic signs of root resorption or periapical radiolucency (Figures 3E–G).

The patient was advised to attend follow-up visits at least once a year until 5 years post-trauma. In addition, the patient was followed up at 7 and 10 years after the injury. At the 5-, 7-, and 10-year follow-up visits, the patient reported no symptoms. Clinical examination revealed the intactness of the composite resin restorations on teeth #32, #31, and #41, with no obvious secondary caries, no tenderness to percussion and no mobility, as well as no obvious abnormalities of the gingiva (Figures 2H–J). Radiographic examination revealed satisfactory root canal fillings in teeth #32, #31, and #41, with clear and continuous PDL spaces and no evidence of root resorption or periapical radiolucency (Figures 3H–J).

### 2.5 Patient perspective

The patient expressed deep anxiety immediately after the injury, fearing the loss of her three anterior teeth and the impact on her appearance and daily life. After detailed discussion, she consented to the PRF-assisted replantation and followed the postoperative instructions. Over the 10-year follow-up, she remained asymptomatic with stable teeth and no root resorption on radiographs, which brought lasting reassurance and satisfaction. In her own words, she “could not be more satisfied with the outcome” and hopes her experience can encourage other patients with similar trauma to consider this effective treatment option that worked well for her.

## 3 Discussion

The extraoral time (the duration for which a tooth remains outside the alveolar socket) is a critical factor determining the prognosis of replanted avulsed teeth (2, 15). Replantation within 5 min can lead to periodontal healing (20). However, for an avulsed tooth under dry storage for more than 5 min, the viability of PDL cells is compromised (21). When replantation is delayed for more than 1 h, the damaged PDL tissue is highly likely to undergo complete necrosis. If the extraoral exposure time is more than 2 h, the risk of external resorption increases significantly, reaching approximately 95% (22). Teeth that develop inflammatory resorption after replantation are typically lost within the first 4 years, whereas those with replacement resorption have a lower risk of loss during the first 3 years, but their loss is still the predominant long-term outcome (23). Coste et al. reported an overall survival rate of only 50% at 5.5 years after replantation of permanent teeth (24, 25).

Before replantation, the storage medium is another critical factor influencing the viability of PDL cells (18, 20). In a 2020 systematic review, De Brier et al. described that media such as Hank's balanced salt solution, propolis, oral rehydration solution, and rice water may be as effective as milk in maintaining the viability of PDL cells (26). In real-world situations, however, owing to the lack of knowledge regarding the early management of dental trauma, few patients are able to undergo immediate replantation or store the avulsed tooth in an ideal medium. Studies have demonstrated that 28.6% of patients store the avulsed tooth in dry tissue paper, and only 11% place the tooth in the mouth during transport to the clinic (27). In the present case report, the patient wiped the root surface of the avulsed teeth with tissue paper, resulting in the detachment of periodontal tissue from the root surface and the complete loss of the tissue that could otherwise have promoted periodontal healing. Subsequently, the teeth were stored dry on tissue paper, and the patient presented to the emergency department more than 2 h after the injury. The extraoral time of more than 2 h had already far exceeded the ideal window for immediate replantation. Thus, the probability of severe root resorption as a complication following direct replantation was extremely high, rendering the treatment of this case significantly more challenging.

PRF, an autologous biomaterial, is prepared through centrifugation without the use of anticoagulants, resulting in a three-dimensional fibrin network enriched with platelets, white blood cells, and various growth factors. This material can continuously release bioactive factors that promote osteoblast proliferation, angiogenesis, and tissue repair (28). Case reports with short-term follow-up have reported that PRF may inhibit root resorption after the replantation of avulsed teeth (14, 29). In the current case report, during the replantation procedure, we utilized PRF granules to promote the healing of the PDL in the avulsed teeth, potentially limit the progression of severe complications such as root resorption, and prolong the retention of the replanted teeh. These outcomes were consistent with our expectations; during follow-up at 3 months, 6 months, 12 months, and even 10 years postoperatively, no signs of root resorption were observed clinically or radiographically in the affected teeth. These findings suggest that the application of PRF may represent a promising adjunctive approach for avulsed teeth, which traditionally have a poor prognosis.

In this case, over a follow-up period of up to 10 years, the three replanted teeth showed no obvious inflammatory or replacement root resorption and achieved favorable PDL healing, suggesting a potential adjunctive role of PRF that warrants further investigation. Most studies on PRF-assisted tooth replantation had follow-up periods of 2 years, but root resorption may have occurred as late as 5 years after replantation (15, 16, 29, 30). The IADT guidelines emphasize the need for long-term monitoring of replanted teeth for the development of late complications (31), and the present case report precisely fulfills this requirement. Over the 10-year follow-up, an intact lamina dura and stable mobility were noted, and no resorptive lesions in the replanted teeth were detected. These findings suggest that the regenerative effect of PRF may be sustained, and that PRF may help reduce the risks of early inflammatory resorption and late replacement root resorption. However, these mechanisms were not directly measured in this case. PRF may exert its effects through the following biologically plausible mechanisms, which are derived from previous experimental studies: (1) Recruitment and differentiation of stem cells (13, 14, 32): PRF releases multiple growth factors, such as PDGF, TGF-*β*, and VEGF. It may activate the PDL cells and stem cells residing in the residual PDL tissue within the socket. It may also recruit host stem cells from the circulation to the injury site. Through these mechanisms, PRF may promote stem cell proliferation and differentiation toward a PDL phenotype while inhibiting osteogenic differentiation, preferentially enhancing the proliferation of fibroblasts and the formation of PDL-like structures and reducing the mobilization of bone matrix cells and osteogenic cells. Ultimately, these processes may decrease the risk of aberrant bone healing between the root and the socket. (2) Maintenance of a long-term regenerative microenvironment: Upon the activation of the platelet *α*-granules in PRF, various growth factors are released continuously over a period of 1−4 weeks. These factors have been reported to increase the mitotic activity of PDL fibroblasts by 20%-37% (15). Thus, PRF may enhance the proliferative capacity and periodontal differentiation potential of target cells, thereby potentially providing sustained support for PDL repair. (3) Synergistic regulation by growth factors: PRF retains diverse, abundant growth factors in physiological proportions, which may help maintain tissue homeostasis and regulate healing and regeneration. (4) Physical barrier: The collagen fibers of PRF may form a barrier between the root and alveolar socket, potentially reducing ankylosis and aberrant resorption-related repair. (5) Regulation of osteoclast activity: PRF may promote osteoprotegerin (OPG) secretion, which inhibits osteoclast formation and activity (33). Thus, PRF may reduce the incidence of external root resorption to a certain extent.

Furthermore, in the present case, an accident caused the avulsion of three mandibular anterior teeth. Most studies have focused on the replantation of maxillary central incisors (3, 4, 29). Mandibular anterior teeth differ anatomically from maxillary teeth with relatively narrower roots and thinner alveolar bone and these differences may influence the prognosis of replantation. The avulsion and replantation of multiple mandibular anterior teeth present particular challenges. The healing of adjacent sockets is interrelated, the biomechanical stresses during mastication are greater, and soft-tissue closure is more difficult. Consequently, multiple-tooth replantation is associated with more challenges than single-tooth replantation (34). Most studies have focused on single-tooth replantation (14, 30, 35), and data on the use of PRF in multiple-tooth replantation are limited. The present case report provides clinical evidence for the replantation of mandibular anterior teeth. The results indicate that PRF can overcome the inherent challenges of mandibular anterior tooth replantation. Thus, the application of PRF can be extended to the management of dental trauma.

This study has several limitations. As a single case report, a control group is lacking, and the findings cannot be generalized to all avulsion cases. In addition, the favorable outcome cannot be attributed specifically to PRF, because multiple components of the treatment, including replantation, root canal treatment, splinting, and follow-up, may have contributed to the result. Moreover, radiographic and clinical observations alone cannot demonstrate periodontal regeneration at a histological level. Although the overall management followed the 2012 IADT guidelines, root canal treatment was initiated approximately 2 weeks after replantation, whereas the guidelines recommend initiation within 7−10 days for a replanted tooth with a closed apex. This delay occurred because the patient did not return for the scheduled follow-up visit. This deviation may have increased the risk of infection-related inflammatory root resorption, although no such resorption was observed during follow-up. Furthermore, the specific molecular mechanisms through which PRF may prevent root resorption have not been explored in this case. Further basic research is warranted for validating these mechanisms. In the future, large-sample cohort studies and controlled trials should be conducted to confirm the efficacy of PRF in preventing root resorption in multiple-tooth avulsion and to determine the optimal application protocol.

## 4 Conclusion

The use of PRF in the delayed replantation of avulsed teeth was followed by asymptomatic, functional teeth with favorable PDL healing over a 10-year follow-up period. As a single case without a comparator, this observation suggests a potential adjunctive role of PRF in promoting periodontal healing and inhibiting root resorption, which warrants further investigation.

## Data Availability

The original contributions presented in the study are included in the article/Supplementary Material, further inquiries can be directed to the corresponding author.
